# Factors associated with non-participation and drop-out in a lifestyle intervention for workers with an elevated risk of cardiovascular disease

**DOI:** 10.1186/1479-5868-6-80

**Published:** 2009-12-01

**Authors:** Iris F Groeneveld, Karin I Proper, Allard J van der Beek, Vincent H Hildebrandt, Willem van Mechelen

**Affiliations:** 1 Department of Public and Occupational Health, EMGO Institute for Health and Care Research, VU University Medical Center, Van der Boechorststraat 7, 1081 BT Amsterdam, The Netherlands; 2 Body@Work, Research Center Physical Activity, Work and Health, TNO-VUmc, Van der Boechorststraat 7, 1081 BT Amsterdam, The Netherlands; 3 TNO Prevention and Care, Department of Physical Activity and Health, Wassenaarseweg 56, 2301 CE Leiden, The Netherlands

## Abstract

**Background:**

Non-response and drop-out are problems that are commonly encountered in health promotion trials. Understanding the health-related characteristics of non-participants and drop-outs and the reasons for non-participation and drop-out may be beneficial for future intervention trials.

**Methods:**

Male construction workers with an elevated risk of cardiovascular disease (CVD) were invited to participate in a lifestyle intervention study. In order to investigate the associations between participation and CVD risk factors, and drop-out and CVD risk factors, crude and multiple logistic regression analyses were performed. The reasons for non-participation and drop-out were assessed qualitatively.

**Results:**

20% of the workers who were invited decided to participate; 8.6% of the participants dropped out before the first follow-up measurement. The main reasons for non-participation were 'no interest', 'current (para-)medical treatment', and 'feeling healthy', and for drop-out they were 'lack of motivation', 'current (para-)medical treatment', and 'disappointment'. Participants were 4.2 years older, had a higher blood pressure, higher total cholesterol, and lower HDL cholesterol than non-participants, and were more likely to report 'tiredness and/or stress' and 'chest pain and/or shortness of breath'. After adjusting for age, most risk factors were not significantly associated with participation. Drop-outs were 4.6 years younger than those who completed the study. The prevalence of smoking was higher among non-participants and drop-outs.

**Conclusion:**

Participants had a worse CVD risk profile than non-participants, mainly because of the difference in age. Non-participants and drop-outs were younger and more likely to be smokers. The main reasons for non-participation and drop-out were health-related. Investigators in the field of health promotion should be encouraged to share comparable information.

**Trial registration:**

Current Controlled Trials ISRCTN60545588

## Introduction

Hundreds of volunteers are usually needed for randomized controlled trials (RCTs) focusing on the promotion of a healthy lifestyle, and sufficient participants need to be recruited in order to find a statistically significant effect of the intervention. However, the first problem that is commonly encountered in the recruitment phase is low response, and prolonging the recruitment phase in order to achieve sufficient power is not always possible. A second problem in the recruitment phase is selection bias [1]. Non-respondents may systematically differ from respondents in certain (health-related or socio-demographic) characteristics, and selection bias may impede generalization of the results to the target population. As soon as the recruitment phase has finished, a third problem arises, i.e. drop-out. In the vast majority of studies, a certain proportion of participants do not complete the study. The estimated number of drop-outs is usually taken into account in the power calculation. However, a point of concern is the possibility of selective drop-out [2-4], i.e. higher attrition in either the intervention group or the control group. Selective drop-out may attenuate or enhance the effects of the intervention.

Since non-response and drop-out may lead to bias, it is important to investigate the differences between participants and non-participants in socio-demographic and health-related characteristics. Previous research has shown that participants are relatively more often female and have a higher level of education [5,6]. There is also a tendency for participants in health promotion trials to be slightly more overweight than non-participants [7,8]. It would also be interesting to know the most common reasons for non-participation and drop-out, so that participation rates can be improved in future studies. In health promotion programs aimed at reducing the risk of cardiovascular disease (CVD) and diabetes, the main reasons for non-participation were 'lack of time', 'financial constraints' [8,9], 'travel problems' [10,11], 'no interest' [12], and perceptions of 'being too old or too unwell' [13]. In some studies, reasons for drop-out have also been identified, i.e. 'health problems unrelated to the study' [14-16], 'lack of time', and 'dissatisfaction' [17].

Several authors underline the importance of reporting participation rates, and the characteristics of participants as well as non-participants. These data have clear implications for the representativeness of the population, and consequently the generalizability of the results [18-20]. A sub-study was performed within the Health under Construction Study, to examine the characteristics of non-participants and drop-outs, as well as their reasons. In the Health under Construction Study, the effectiveness of a six-month lifestyle intervention for male construction workers with an elevated CVD risk was evaluated. The intervention, provided by occupational physicians and nurses, consisted of individual counseling based on motivational Interviewing, encouraging participants to stop smoking or to increase physical activity and/or to improve their dietary behavior. Three face-to-face contacts with a duration of 45-60 minutes, and four telephone conversations, each lasting 15-30 minutes, were scheduled for each participant. The design and inclusion criteria of the study have been described more extensively elsewhere [21]. The study was commissioned by Arbouw, the Dutch national organization involved in monitoring and improving labor conditions and occupational health of workers in the construction industry. The Medical Ethics Committee of the VU University Medical Center approved the study protocol.

In this paper we describe: 1) recruitment, participation, and drop-out rates; 2) reasons for non-participation and drop-out; 3) differences in age and CVD risk-related characteristics between participants and non-participants, and between drop-outs and participants who completed the first follow-up measurement.

## Methods

### Invitation procedure

Each month, all Dutch occupational health services (OHSs) provide Arbouw with the results of the most recent periodical Health Risk Appraisals (HRAs) of workers in the construction industry. The results include CVD risk-related variables, and these data were used to select workers who were at risk for CVD, by applying a predefined screening instrument. All eligible workers, those with an elevated risk according to the screening instrument, were invited by Arbouw to participate in the Health under Construction Study. In order to guarantee anonymity, Arbouw coded all HRA data before sending them to the researchers.

Based on a power calculation, the minimum number of participants needed to detect a 10% difference between groups in meeting the Dutch guidelines for moderate intensity physical activity was 692. Anticipating a drop-out of 20%, 865 workers should have been included. Male construction workers, aged 18-55 years, with an elevated CVD risk, were invited to participate. For logistical reasons, only workers in certain predefined geographical areas in The Netherlands were invited. Due to a low response, after five months the inclusion criteria were adjusted. The maximum age was extended to 65 years, and to all male construction workers in The Netherlands with an elevated CVD risk. Each invited worker received a letter and a brochure explaining the study, describing the benefits (better health, two free health check-ups, and a chance to win a three-day holiday), and the importance of improving future occupational health care. The safety of the study was emphasized, as well as the fact that the intervention would take place outside working hours at the nearest OHS. Included in the invitation were a six-page questionnaire on lifestyle, absenteeism, medication use, and subjective health, and an informed consent form. By signing this consent form, the worker confirmed that he was aware of the 0.5 chance of randomization to the control group, and that he agreed to undergo the physical health check-ups and complete the follow-up questionnaires after 6 and 12 months.

### Data-collection

The invited workers were asked to return the signed consent form and the questionnaire in the envelope that was provided, but also to send the consent form back even if they had decided *not *to participate, and to give their reasons for non-participation. Workers who did not respond to the first invitation within three weeks received a reminder, accompanied by the questionnaire and the consent form. Those who did not send the consent form back within a month after the second invitation were considered to be non-respondents. Drop-out was notified in one of four ways: by the worker himself, his wife, a counselor, or a medical assistant at the OHS. If a participant in the intervention group had dropped out without any explanation, he was not asked to give his reason, but he was asked by telephone to complete the follow-up questionnaire and to attend the follow-up health check-up. During this telephone call, any misunderstandings were clarified, and some workers reconsidered their decision. In case of 'no show' at the follow-up health check-up, the participant was phoned to make a new appointment. Participants who did not return the follow-up questionnaire were phoned within a month by one of the researchers and asked to do so. Participants who did not attend the physical health check-up, did not send the questionnaire back, and did not respond to telephone calls, were defined as 'drop-outs without reason'. A participant who only attended the health check-up or only sent the questionnaire back was not considered as a drop-out. The procedure for clustering reasons for drop-out was comparable to that for clustering reasons for non-participation.

During the periodical HRA, six biological CVD risk factors, i.e. body weight, systolic blood pressure (SBP), diastolic blood pressure (DBP), total cholesterol, HDL cholesterol, and hemoglobin A1c (HbA1c), were measured according to the HRA protocol. The HRA also included a questionnaire: Two items were related to heart problems, i.e. 'occasionally suffering from chest pain' and 'occasionally suffering from shortness of breath'. 'Chest pain and/or shortness of breath' was confirmed if one or both items were scored positively. Twelve items related to psychological risk factors, grouped in two clusters, i.e. 'tiredness', and 'ability to cope with work demands'. 'Tiredness and/or stress' was confirmed if more than 5 out of the 12 items were scored positively. Lifestyle-related CVD risk factors were also assessed by the questionnaire, i.e. smoking (yes/no), and not meeting the Dutch guidelines for moderate intensity physical activity (at least 5 days a week for a minimum of 30 minutes a day ()[22]) or for vigorous intensity physical activity (at least 3 days a week for a minimum of 20 minutes a day [23])'. Since type of work (administrative and supervisory tasks vs. construction tasks) can be regarded as a proxy for physical activity at work (little or none at all vs. a lot), type of work was also assessed.

### Data analysis

We calculated the percentages of non-respondents, respondents who had agreed to participate, and respondents who had not (the latter will be referred to as 'non-participants-with-reason' [NPWR] in the remainder of this article). To make interpretation easier we clustered reasons that were inter-related, based on common sense. Some NPWR gave more than one reason, and because all reasons were recorded, the total number of reasons exceeded the number of NPWR. For each cluster of reasons, we calculated the proportion of the total number of reasons. The procedure for clustering reasons for drop-out was comparable to that for clustering reasons for non-participation.

For both participants and non-participants, means and standard deviations were presented for the continuous variables: age, BMI, SBP, DBP, total cholesterol, HDL cholesterol, and HbA1c. Percentages were presented for the dichotomous variables: smoking (yes/no), 'chest pain and/or shortness of breath' (yes/no), 'tiredness and/or stress' (yes/no), 'meeting none of the two Dutch physical activity guidelines' (yes/no), and type of work (administrative and supervisory tasks vs. construction tasks). In order to investigate the association between each variable and participation, crude regression analyses were performed with participation as the dependent variable. Subsequently, a multiple logistic regression model was constructed to investigate the associations adjusted for other variables. A variable that had a p-value < 0.05 in the multiple regression model was considered to be significantly associated with participation. The model was built using a forward stepwise procedure, starting with the variable with the lowest p-value in the crude analysis, followed by the next lowest, and so on. Only variables with a p-value < 0.1 in the crude analysis were tested for association. To obtain further insight into the association between age and participation, three age groups were defined (30-39, 40-49, and 50-65 years) and compared to a reference category (18-29 years), by calculating the Mantel Haenszel odds ratios (ORs). Identical analyses were performed with drop-out as the dependent variable.

## Results

### Response and reasons

**Figure 1:**
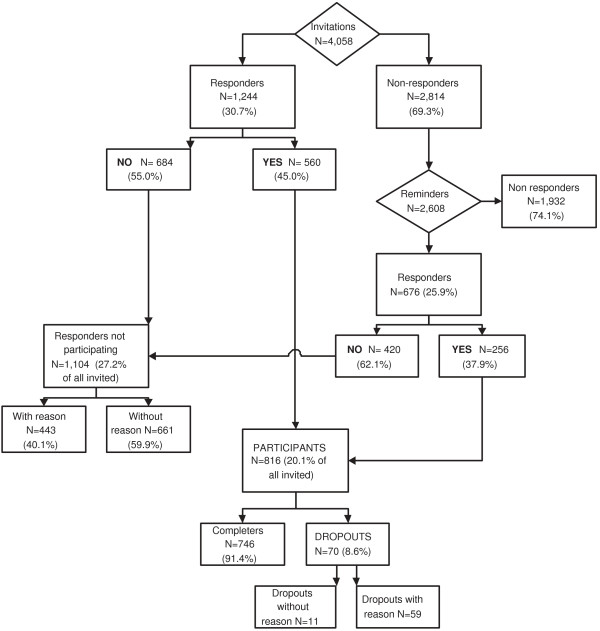
**Flow-chart of inclusion and drop-out**.

Figure 1 presents a flow-chart of inclusion and drop-out. Of the 4,058 invited workers who were invited, 30.7% sent the consent form back. Those who did not received a reminder, to which 25.9% responded. In total, 1,104 (27.2% of all workers invited) were unwilling to participate, 443 (40.1%) of whom gave one or more reasons. Of all the participants, 70 (8.6%) dropped out before the first follow-up measurement (47 [67.1%] were allocated to the intervention group), 59 of whom reported their reasons for drop-out.

Table 1 shows the reasons for non-participation and drop-out; the main reasons for both were 'not motivated' or 'not interested'. 'Having other health problems' and 'already receiving medical treatment' were also frequently mentioned by both NPWR and drop-outs. In most cases, it was not clear whether this treatment was aimed at CVD or at some other health problem. 'Feeling healthy', 'already adopted a healthier lifestyle', or 'the regular HRA is sufficient' were reasons that were only given by non-participants. Of the NPWR, 13 did not want to participate because they did not trust the OHS, and 42 stated that they were no longer working in the construction industry or would be leaving in the near future, due to a change of job or retirement. The reason frequently reported by drop-outs in the intervention group was 'disappointment', mainly in the organization, e.g. due to a change of counselor or inaccurate planning of counseling appointments. The OHSs had failed to schedule the health check-ups for 8 participants, and because they had not sent their questionnaire back, they were considered as drop-outs.

**Table 1:** Reasons for non-participation and drop-out in the Health under Construction Study.

Reasons	Cluster	NPWR		Drop-outs	
		(N)	(%)	(N)	(%)
Feeling healthy; already adopted/starting to adopt a healthier lifestyle; regular HRA is sufficient	Health	88	19.0	-	-
Dissatisfied with the OHS or not trusting the project	Distrust	13	2.8	-	-
No interest; not motivated	Motivation	104	22.5	17	28.8
					
Other health problems/currently receiving (para-) medical treatment	Treatment	96	20.8	11	18.6
Lack of time; expenses too high; travel distance too far	Time/Money	84	18.2	9	15.3
Retired or working in a different branch	Work	42	9.1	2	3.4
Other, e.g. personal reasons	Other	35	7.6	3	5.1
					
Disappointed in organization e.g. due to counselor change	Disappointment	-	-	9	15.3
Follow-up measurements planned too late or not at all	Organization	-	-	8	13.6

Total reasons		462	100	59	100

NPWR: non-participants-with-reason. HRA: periodical health screening, OHS: occupational health service

### Characteristics and associations

Table 2 presents the characteristics of participants and non-participants. The participants were older, had higher SBP, DBP and total cholesterol, and lower HDL cholesterol, and were less likely to smoke. With respect to type of work, fewer construction workers participated, compared to workers involved in administration and supervision. In the crude logistic regression models, the variables age, DBP, total cholesterol, HDL cholesterol, 'chest pain and/or shortness of breath', 'tiredness and/or stress', smoking, and type of work were significantly associated with participation. In the multiple logistic regression model, only age, smoking, type of work, and 'chest pain and/or shortness of breath', remained statistically significant. SBP also appeared to be significantly associated with participation, although in the opposite direction to that in the crude model. Of all the variables, age appeared to have the strongest association with participation (OR 1.04; 95% confidence interval [CI] 1.03-1.05), and could be regarded as a confounder in the relationship between participation and most other variables. The characteristics of drop-outs and non-drop-outs are presented in Table 3. Of all the drop-outs, 61.4% had been smokers, as opposed to 53.5% of the non-drop-outs. In both the crude and the multiple logistic regression model, only age was significantly associated with drop-out; drop-outs were significantly younger than non-drop-outs (OR 0.96; 95%CI 0.93-0.98). The ORs of participation and drop-out in the three different age groups are shown in Table 4.

**Table 2:** Characteristics of participants and non-participants in an individual lifestyle intervention trial for workers at risk for cardiovascular disease, and the associations with participation.

	Participants		Non-participants		Crude	Multiple
	**N**	**Mean** **(sd)**	**N**	**Mean** **(sd)**	**OR** **(95% CI)**	**OR** **(95% CI)**
Age	816	46.08 (9.32)	3,240	42.24** (10.89)	1.04 (1.03;1.04)**	1.04 (1.03; 1.05)**
BMI (kg/m^2^)	816	28.53 (3.61)	3,231	28.29 (4.12)	1.01 (1.00; 1.03)	-
SBP (mmHg)	816	142.13 (15.81)	3,231	141.48 (16.68)	1.00 (1.00; 1.01)	0.99 (0.99; 1.00)**
DBP (mmHg)	816	88.51 (9.68)	3,231	87.07** (10.10)	1.01 (1.01;1.02)**	-
						
Total cholesterol (mmol/l)	815	6.21 (0.94)	3,222	6.07** (0.98)	1.16 (1.07;1.25)**	-
HDL cholesterol (mmol/l)	809	1.12 (0.21)	3,194	1.15** (0.22)	0.58 (0.41;0.84)**	-
HbA1c (%)	810	5.66 (0.40)	3,188	5.63 (0.44)	1.14 (0.96; 1.36)	-
	**N**	**%**	**N**	**%**	**OR** **(95% CI)**	**OR** **(95% CI)**
Smoking (% yes)	809	54.4	3,218	62.7	0.71 (0.61;0.83)**	0.66 (0.56; 0.78)**
Chest pain and/or shortness of breath (% yes)	809	32.5	3,210	28.0	1.24 (1.05; 1.46)*	1.21 (1.02; 1.44)**
Tiredness and/or stress (% yes)	812	35.7	3,205	30.5	1.27 (1.08;1.49)**	-
Fulfilling none of the PA guidelines (% yes)	798	40.7	3,180	40.2	1.02 (0.97; 1.20)	-
Type of work (% administrative/supervisory tasks)	816	23.8	3,240	17.9	0.70 (0.58;0.84)**	0.70 (0.56; 0.78)**

*p < 0.05. **p < 0.01.

sd: standard deviation; OR: odds ratio; CI: confidence interval; BMI: body mass index; SBP: systolic blood pressure; DBP: diastolic blood pressure; HDL: high density lipid; HbA1c: hemoglobin A1c; PA: physical activity

**Table 3:** Characteristics of drop-outs and non-drop-outs in an individual lifestyle intervention trial for workers at risk for cardiovascular disease, and the associations with drop-out.

	Drop-outs		Non-drop-outs		Crude	Multiple
	**N**	**Mean** **(sd)**	**N**	**Mean** **(sd)**	**OR** **(95% CI)**	**OR** **(95% CI)**
Age (years)	70	41.90 (9.79)	745	46.52 (9.12)	0.95** (0.93; 0.98)	0.95** (0.93; 0.98)
BMI (kg/m^2^)	70	28.27 (3.84)	746	28.55 (3.59)	0.98 (0.91; 1.04)	-
SBP (mmHg)	70	142.13 (17.91)	746	142.16 (15.59)	1.00 (0.98; 1.02)	-
DBP (mmHg)	70	87.57 (10.92)	746	88.62 (9.55)	0.99 (0.96; 1.01)	-
Total cholesterol (mmol/l)	69	6.17 (0.90)	746	6.22 (0.94)	0.95 (0.73; 1.24)	-
HDL cholesterol (mmol/l)	69	1.14 (0.21)	741	1.12 (0.21)	1.67 (0.52; 5.37)	-
HbA1c (%)	69	5.62 (0.41)	742	5.67 (0.44)	0.79 (0.43; 1.47)	-
						
	**N**	**%**	**N**	**%**	**OR** **(95% CI)**	**OR** **(95% CI)**
Smoking (% yes)	69	61.4	740	53.5	1.44 (0.87; 2.39)	-
Chest pain and/or shortness of breath (% yes)	69	35.7	743	32.0	1.21 (0.72; 2.01)	-
Tiredness and/or stress (% yes)	70	34.3	746	35.8	0.94 (0.56; 1.57)	-
Fulfilling none of the PA guidelines (% yes)	67	41.4	731	40.4	1.13 (0.68; 1.87)	-
						
Type of work (% administrative/supervisory tasks)	70	24.3	746	23.6	0.96 (0.54; 1.71)	-

**p < 0.01.

sd: standard deviation; OR: odds ratio; CI: confidence interval; BMI: body mass index; SBP: systolic blood pressure; DBP: diastolic blood pressure; HDL: high density lipid; HbA1c: hemoglobin A1c; PA: physical activity.

**Table 4:** The odds ratios of participation and drop-out for three different age groups in a lifestyle intervention trial for workers at risk for cardiovascular disease.

Variable	Participation OR (95% CI)	Drop-out OR (95% CI)
Age 30-39 (reference age: 18-29)	1.64** (1.16 - 2.32)	0.91** (0.38 - 2.21)
Age 40-49 (reference age: 18-29)	2.69** (1.98 - 3.66)	0.45** (0.20 - 1.01)
Age 50-65 (reference age: 18-29)	3.30** (2.43 - 4.48)	0.27** (0.11 - 0.63)

** P < 0.01

OR: odds ratio; CI: confidence interval.

## Discussion

Of the workers who were invited, 20% participated in this lifestyle intervention trial. The reasons for non-participation were related to 'current (para-)medical treatment', 'feeling healthy', and 'no interest'. The participants were significantly older than the non-participants and, mainly related to this age-difference, their CVD risk profile was worse than that of the non-participants. However, smoking was negatively related to participation, irrespective of age. Relatively few participants dropped out before the first follow-up measurement. The reasons given for drop-out were 'no interest', 'current (para-)medical treatment' and 'disappointed in the organization'. Drop-outs were generally younger and more likely to smoke, but their CVD risk profile did not differ significantly from that of the non-drop-outs

Even though the Health under Construction Study is not the only workplace lifestyle intervention study with a low participation rate [24,25], many other such intervention studies had a participation rate of far more than 20% of the target group [17,26-28]. Sending invitations by post to the home address of individual workers, and not involving their employer or colleagues, may partly explain the low response in our study. Drop-out in our study remained lower than in several other workplace intervention studies involving lifestyle counseling [17,25,27].

Despite the fact that non-participants gave reasons such as 'feeling healthy', most of them still had high cholesterol levels and/or high blood pressure. It is possible that their risk perception was inadequate. In several studies a substantial mismatch between actual and perceived risk has been found [29-32], partly caused by insufficient knowledge. In the invitations we sent, we could have specified and explained the individual risk profile. A second important reason for non-participation was 'already receiving (para-) medical treatment'. One way to address this issue would have been to explain, in the invitation letter or brochure, the possible additional positive effect of changes in lifestyle on their current (pharmacological) treatment. Finally, almost 10% of the workers who were invited appeared to have switched between various sectors of industry, or to have retired recently. This could not easily be avoided, and nor could lack of time, motivation, or external reasons. In conclusion, some, but not all of these problems might have been prevented. However, it is questionable whether participation rates could have been increased in any way; there might have been underlying motives that were not known to us. Inevitably, we had to rely on the data reported by the participants themselves. Likewise, for dropout, some but not all problems might have been prevented. Clearly, planning of counseling sessions and health check-ups should have been accurate to prevent disappointment and involuntary drop-out. For a considerable number of drop-outs 'time constraints' appeared to be a problem. By scheduling telephone contacts instead of face-to-face contacts, or by finding an OHS located more closely to their residence or workplace, we were able to solve this problem for some participants. Again, some of the above-mentioned reasons may have been related to a lack of motivation.

Older workers were more willing to participate. Not surprisingly, when adjusting for age, the association between participation and some important CVD risk factors, e.g. total and HDL cholesterol, was no longer significant [33]. Older workers were not only more likely to participate but also to complete the study, a finding that is in line with several other trials [26,34,35]. However, the differences between drop-outs and non-drop-outs in CVD risk factors were only small. This is surprising, since the CVD risk-related variables would be expected to worsen with age. It should be noted that for some workers, drop-out or completion of the study may have been related to (a change in) lifestyle or CVD risk factors. Overall, there was a mismatch in age and CVD risk factors between participants and non-participants, as well as between drop-outs and non-drop-outs. Apparently, the lifestyle intervention was applied only to a sub-group of the target population.

A strength of the study is that we systematically studied the reasons for non-participation of more than 400 NPWR. Furthermore, because we obtained HRA data of all non-participants, we were able to analyze the differences in CVD risk factors between participants and non-participants, including the non-respondents. The information generated in this study may be beneficial for the development of future prevention trials. However, some limitations should also be mentioned. First of all, we did not know the reasons for non-participation of more than half of the workers who were invited. The reasons reported by the NPWR may not have been the same as those of the non-respondents. Secondly, the definition of drop-out may not have been accurate, because we expected that some drop-outs would complete the second follow-up measurement. Thirdly, not all of the results, and in particular, the data on population characteristics, can be generalized to other study populations.

In our study, the response rate was relatively low and only a subgroup of the target population participated and completed the study. Based on these findings, we would recommend that for future interventions investigators should 1) make a realistic calculation of the number of participants needed and the number of persons that need to be invited; 2) anticipate possible reasons for non-participation in a pilot study of the target population, and take into consideration our proposed solutions for stimulating participation and preventing drop-out; 3) adjust the recruitment strategy in order to include the entire target population.

This is one of the few studies in which characteristics, as well as reasons for non-participation and drop-out, in a lifestyle intervention study were systematically investigated. We learned that an elevated CVD risk was positively associated with participation, but that this association was mainly due to age. Reasons for non-participation and drop-out were mainly related to perceived health, current treatment and lack of motivation. In future studies, some of these problems could be anticipated, thereby increasing participation and completion rates. Investigators in the field of health promotion are encouraged to share comparable information, so that by learning from each other's experiences, intervention studies can be performed more efficiently and yield more valid results.

## Competing interests

The authors declare that they have no competing interests.

## Authors' contributions

IG collected the data, performed the data-analyses and wrote the manuscript. KP initiated the Health under Construction Study and contributed to the data-analyses. All authors provided intellectual input and contributed to the writing of the manuscript, and read and approved the final manuscript.
